# Multiple upstream modules regulate zebrafish *myf5 *expression

**DOI:** 10.1186/1471-213X-7-1

**Published:** 2007-01-03

**Authors:** Yau-Hung Chen, Yun-Hsin Wang, Min-Yen Chang, Cheng-Yung Lin, Chih-Wei Weng, Monte Westerfield, Huai-Jen Tsai

**Affiliations:** 1 Graduate Institute of Life Sciences, Tamkang University, Tamsui, Taiwan; 2 Institute of Molecular and Cellular Biology, National Taiwan University, NO. 1, Roosevelt Road, Sec. 4, Taipei 106, Taiwan; 3 Institute of Neuroscience, University of Oregon, Eugene, OR, 97403, USA

## Abstract

**Background:**

Myf5 is one member of the basic helix-loop-helix family of transcription factors, and it functions as a myogenic factor that is important for the specification and differentiation of muscle cells. The expression of *myf5 *is somite- and stage-dependent during embryogenesis through a delicate regulation. However, this complex regulatory mechanism of *myf*5 is not clearly understood.

**Results:**

We isolated a 156-kb bacterial artificial chromosome clone that includes an upstream 80-kb region and a downstream 70-kb region of zebrafish *myf5 *and generated a transgenic line carrying this 156-kb segment fused to a green fluorescent protein (GFP) reporter gene. We find strong GFP expression in the most rostral somite and in the presomitic mesoderm during segmentation stages, similar to endogenous *myf5 *expression. Later, the GFP signals persist in caudal somites near the tail bud but are down-regulated in the older, rostral somites. During the pharyngula period, we detect GFP signals in pectoral fin buds, dorsal rostral myotomes, hypaxial myotomes, and inferior oblique and superior oblique muscles, a pattern that also corresponds well with endogenous *myf5 *transcripts. To characterize the specific upstream *cis*-elements that regulate this complex and dynamic expression pattern, we also generated several transgenic lines that harbor various lengths within the upstream 80-kb segment. We find that (1) the -80 kb/-9977 segment contains a fin and cranial muscle element and a notochord repressor; (2) the -9977/-6213 segment contains a strong repressive element that does not include the notochord-specific repressor; (3) the -6212/-2938 segment contains tissue-specific elements for bone and spinal cord; (4) the -2937/-291 segment contains an eye enhancer, and the -2937/-2457 segment is required for notochord and myocyte expression; and (5) the -290/-1 segment is responsible for basal transcription in somites and the presomitic mesoderm.

**Conclusion:**

We suggest that the cell lineage-specific expression of *myf5 *is delicately orchestrated by multiple modules within the distal upstream region. This study provides an insight to understand the molecular control of *myf5 *and myogenesis in the zebrafish.

## Background

Members of the basic helix-loop-helix (bHLH) family of transcription factors, such as Myf5, MyoD, Myogenin, and MRF4, are crucially important in the specification and differentiation of skeletal muscle progenitors [1]. These myogenic regulatory factors (MRFs) activate muscle-specific transcription by binding to an E-box in the promoter of numerous muscle-specific genes [2,3]. MRF genes are expressed in zebrafish somites in a characteristic temporal sequence, with *myf5 *at 7.5 hours postfertilization (hpf) [4], *myod *at 8 hpf [5], and *myogenin *at 10.5 hpf [5]. The same temporal sequence occurs in mice [1]. These observations indicate that *myf5 *is the first MRF expressed during vertebrate myogenesis.

Mechanisms that lead to Myf5 activation at multiple sites in mouse embryos have been described [6,7]. Yeast artificial chromosomes (YAC) [6] and bacterial artificial chromosomes (BAC) [7] have been used to map the promoter of mouse *myf*5, suggesting that several different *cis-*regulatory elements are required to activate *myf*5 expression in different cells at different developmental times. An enhancer at -6.6 kb is required for *myf*5 expression in the epaxial domain [8]. A 270-bp core enhancer at -57 kb directs *myf*5 expression in limbs and maintains *myf*5 expression in somites [9]. In *Xenopus*, two negative regulatory elements have been identified: an interferon regulatory factor-like DNA binding element that down-regulates *Xmyf5 *expression in differentiating myocytes [10], and a distal TCF-3 binding site by which Wnt/β-catenin signaling restricts *Xmyf5 *expression to the midline mesoderm [11]. A T-box binding site mediates dorsal activation of *Xmyf5 *transcription and is involved in the regulation of muscle development [12]. Using transient expression of transgenes, we previously identified some *cis*-elements that regulate zebrafish *myf5 *[4,13,14]. Recently, Lee *et al*. [15] demonstrated that Foxd3 binds to the -82/-62 regulatory module and regulates zebrafish *myf5 *expression during early somitogenesis. These observations highlight the complicated and dispersed nature of the upstream elements that control somite- and stage-specific expression of *myf5*.

To elucidate the nature of this finely tuned control mechanism, we needed a transgenic line that recapitulates the specific endogenous expression pattern of *myf5*. Such a line requires a transgene that contains a very long upstream region of *myf5*. We modified the techniques used in mice [16,17] and present here a highly efficient method for engineering zebrafish BAC. The BAC is an *Escherichia coli *F factor-based vector that is capable of propagating cloned DNA fragments up to 300 kb long [18]. Previously, Jessen *et al*. [19] reported a homologous recombination technique for BAC cloning to generate transgenic zebrafish. This technique, however, is rather laborious because it requires a chi-based plasmid with a very large recombination targeting region. With our new method, we efficiently generate transgenic lines containing a 156-kb genomic sequence of *myf5 *(80-kb upstream and 70-kb downstream segments) replaced with green fluorescent protein (GFP) in the coding region. We find that the 156-kb genomic sequence is long enough to recapitulate the endogenous *myf5 *transcription pattern during the somitogenesis and pharyngula development stages. To characterize the functions of individual *cis*-regulatory elements, we also generated several transgenic lines that carry various lengths of the zebrafish *myf5 *upstream sequence. Comparing the GFP expression patterns of these lines, we identified and characterized the functions of upstream regulatory elements, including a repressive element and tissue-specific enhancers for jaw and fin muscles, bones, eyes, somites, olfactory organs, and the presomitic mesoderm. Whole-mount *in situ *hybridization reveals that endogenous zebrafish *myf5 *transcripts are first detectable in the presomitic mesoderm [4]. In contrast, *myf5 *expression has not been observed by *in situ *hybridization in the presomitic mesoderm of mouse embryos, although weak signals have been detected by reverse transcriptase-polymerase chain reaction (RT-PCR) [20,21]. These comparisons illustrate the advantage of our transgenic lines for studying the initiation and regulation of *myf5*, particularly in the presomitic mesoderm.

## Results

### Genomic organization of zebrafish *myf5 *is conserved with mammals

We cloned the *myf5 *genomic locus using a sequential screening method. We screened 10 primary pools of zebrafish BAC library clones using PCR; one (P1) was positive. We then screened 48 secondary pools derived from P1 and found 5 positive pools. Subsequent screening of the positive pools identified a single *myf5*-positive BAC clone with a 156-kb insert as indicated by PFGE. We searched sequence databases from the Sanger Institute and identified a contig, ctg9418, which contains the entire sequence of the *myf5 *BAC clone. BLAST analysis using the *myf5 *coding region and junction sequences flanking the T7 and SP6 sites showed that the BAC clone spans the sequences of ctg9418 from 1460 to 1304 kb, with the coding sequence starting at 1380. Thus, the *myf5 *5' and 3' regions contained in this BAC clone extend approximately 80 kb and 70 kb, respectively, beyond the coding sequence.

Radiation hybrid mapping of zebrafish *myf5 *revealed that *myf5 *is located on linkage group 4 (LG4), between expressed sequence tags (ESTs) fb62d08 and fb78c03, and at 5.87 CR from EST marker z9667 (data not shown). This syntenic relationship indicates that the region of zebrafish LG4 between EST markers fa05f06 and fk68a09 (including *myf5*) is conserved with human chromosome 12q13–12q21.

### DNA fragments of *myf5*:*GFP *are inherited

**Figure 1:**
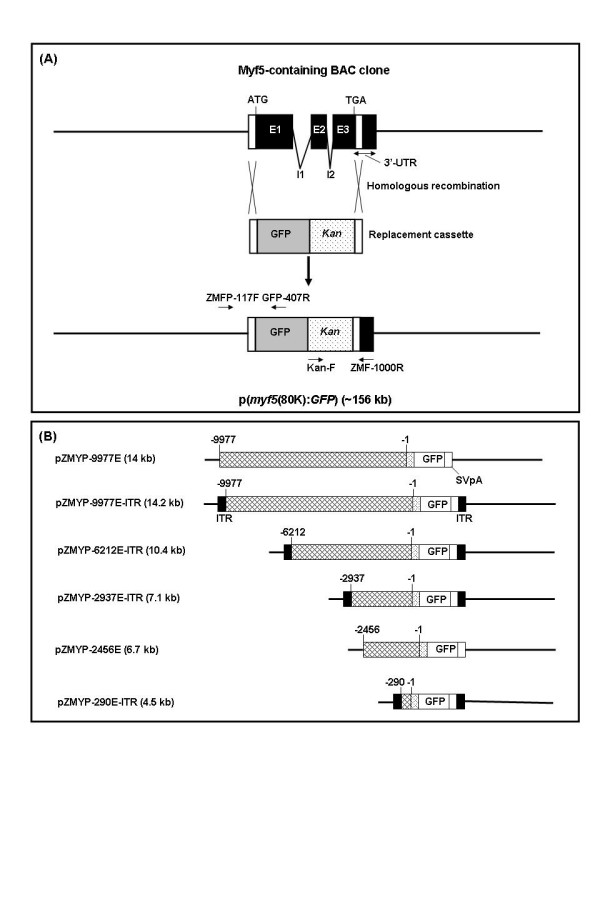
**Construction of a *myf5:GFP *bacterial artificial chromosome (BAC) and deletion constructs for germ-line transmission in zebrafish**. (A) Strategy for constructing a *myf5 *BAC clone containing the green fluorescent protein (GFP) reporter. (Top) The genomic organization of the *myf5 *that contains 3 exons (E1, E2, and E3) and 2 introns (I1 and I2). (Bottom) The resulting p(*myf5*(80K):*GFP*) clone contains the *myf5 *upstream 80-kb regions fused with the GFP reporter gene. The primers ZMFP-117F, GFP-R, Kan-F, and ZMF-1000R were used to check recombinants. (B) Deletion constructs used in this study. Plasmid pZMYP-2456E was described by Wang *et al*. [13]. Thick lines and crossed boxes represented plasmid vectors and *myf5 *promoters, respectively. Numbers above the boxes indicate the nucleotide positions relative to the transcription start site of zebrafish *myf5*. GFP, green fluorescent protein; ITR, inverted terminal repeats of adeno-associated virus; SVpA, polyadenylation signal of SV40.

We generated several transgenic lines by microinjecting zebrafish embryos with *myf5*:*GFP *BAC segment and raising them to adulthood. We identified germ-line transmission by looking for GFP fluorescence in embryos from crossing with wild-type fish. We generated 4 lines with the entire *myf5 *upstream segment (the 156-kb group: 80k-5, -18, -21, and -23; Fig. 1A), 4 lines with -9977/-1 (10k-2, -13, -9R, -15R; Fig. 1B), 4 lines with -6212/-1 (6k-9R, -10R, -11R, -16R; Fig. 1B), 3 lines with -2937/-1 (3k-18R, -92R, -104R; Fig. 1B), 3 lines with -2456/-1 (2.4k-3, -8, -55; Fig. 1B), and 2 lines with -290/-1 group (0.3k-14R, -112R; Fig. 1B). The F2 segregation frequencies for these transgenic lines ranged from 47.5 to 52.9%, indicating a single insertion site of transgene for each line.

### The 156-kb genomic sequence of *myf5 *drives GFP expression in muscle precursors

**Figure 2:**
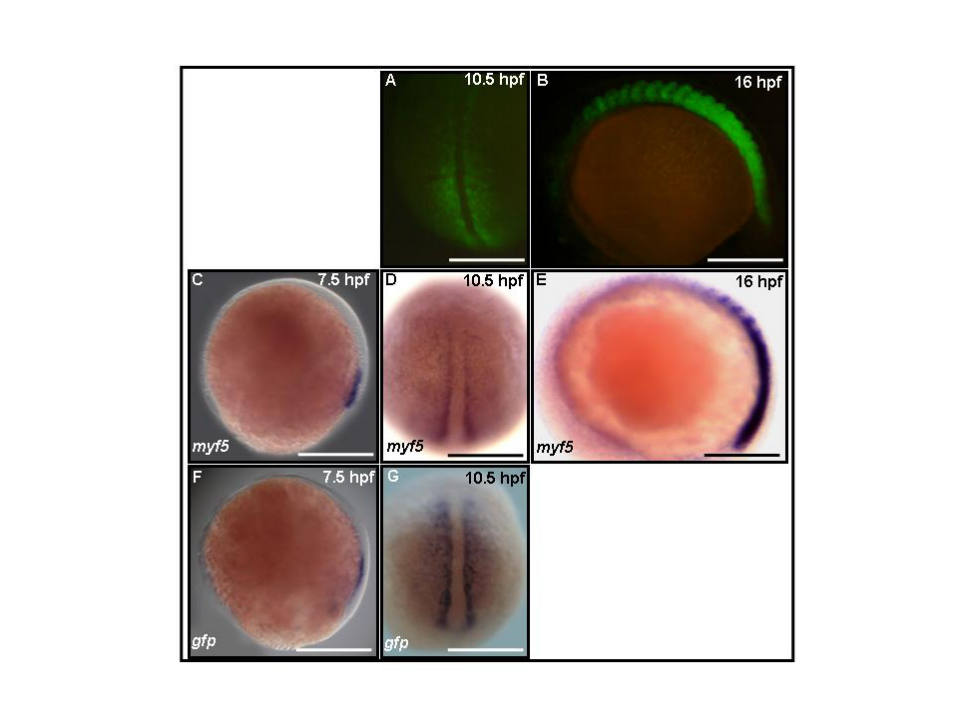
**Green fluorescent protein (GFP) expression in *Tg*(*myf*5(80K):GFP) transgenic embryos recapitulates endogenous *myf5 *expression in muscle precursors**. GFP fluorescence is detected in the presomitic mesoderm of embryos by 10.5 hours postfertilization (hpf) (A), in the somites and the presomitic mesoderm in 16 hpf embryos (B). Endogenous *myf5 *transcripts (C,D,E) and GFP mRNA (F,G) were detectable at 7.5, 10.5, and 16 hpf. (A,D,G) Dorsal views, rostral to the left; (B,C,E,F) side views, rostral to the left, dorsal to the top. Scale bars: 100 μm in all panels.

In embryos derived from *myf5*:*GFP *transgenic lines with the 156-kb genomic sequence (an upstream 80-kb segment and a downstream 70-kb segment; Fig. 1A), GFP fluorescence first appears very weakly at 7.5 hpf (data not shown), reaches detectable levels in the segmental plate by 10.5 hpf (Fig. 2A), and expands to 14 somite pairs in 16 hpf embryos (Fig. 2B). At 16 hpf, the GFP signals are strong and mainly restricted to the somites and segmental plates and fluorescence is reduced in the more rostral (older) somites. Prominent GFP signals also appear in the adaxial cells (Fig. 2B). The GFP mRNA in embryos derived from *myf5*:*GFP *transgenic lines were also detected by using digoxigenin (DIG)-labeling GFP riboprobes. The GFP transcripts were detectable at 7.5 hpf (Fig. 2F) and 10.5 hpf (Fig. 2G), which matches the spatial and temporal pattern of endogenous *myf5 *expression as indicated by mRNA *in situ *hybridization (Figs. 2C–2E) [4]. These results indicate that expression of GFP in the *myf5*:*GFP *transgenic line recapitulates endogenous *myf5 *expression.

**Figure 3:**
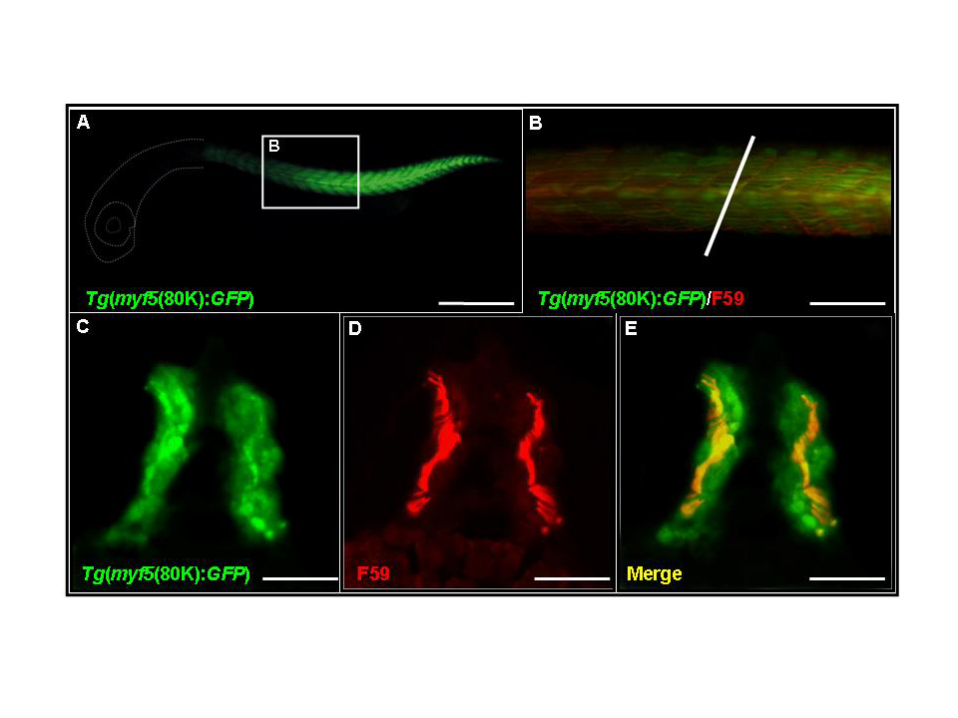
***Tg*(*myf*5(80k):GFP) transgenic embryos express green fluorescent protein (GFP) in both slow and fast muscle fibers**. (A,B) GFP expression in somites labeled with the F59 antibody. White dash lines indicate the location of head. (B) Higher magnification view of the boxed region shown in A. (C-E) Cross-section along the plane indicated by the white line in panel (B). GFP signals are observed in both fast (C, green signals) and slow muscle fibers (F, yellow signals). (A,B) Side views, rostral to the left, dorsal to the top; (C-D) dorsal to the top. 28 hpf. Scale bars: 400 μm in A; 200 μm in B; 100 μm in C-E.

By 28 hpf, GFP fluorescence is absent from rostral (older) somites (Figs. 3A and 3B). Cross-sections reveal that GFP-positive cells are distributed throughout the myotome (Fig. 3C). We use the F59 antibody to label slow muscle fibers and find that both slow and fast muscle fibers express GFP in the *myf5*:*GFP *transgenic line (Figs. 3D and 3E). In addition, we find no significant differences when comparing the GFP expression patterns of the four independent *myf5*:*GFP *lines (80k-5, -18, -21, and -23), indicating that expression of the transgene is unaffected by its chromosomal location.

### GFP expression reveals the development of head skeletal muscles

**Figure 4:**
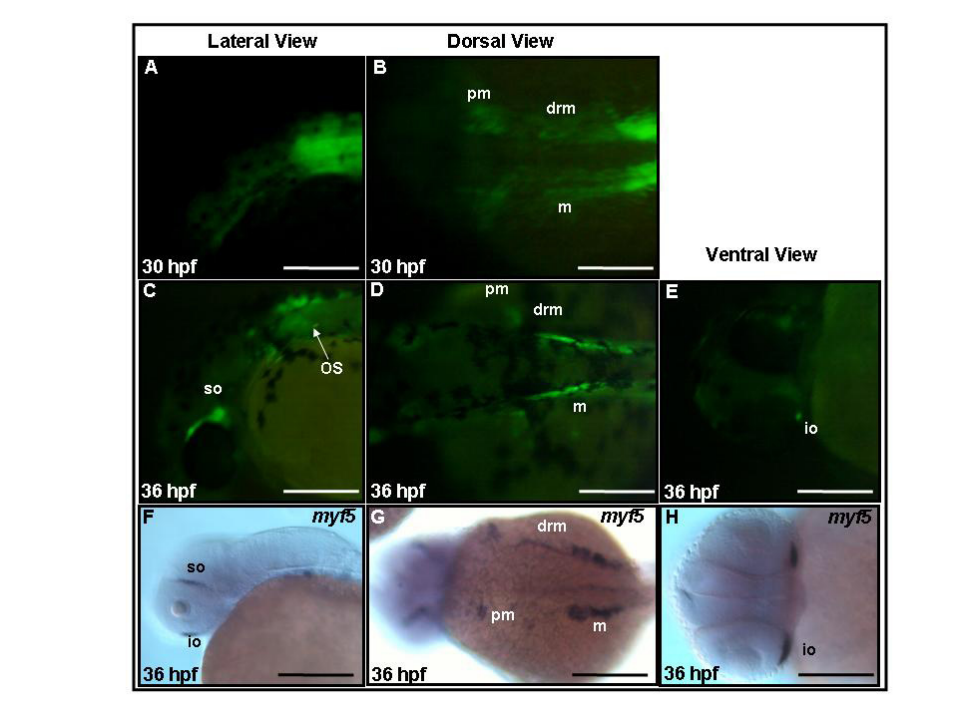
**Expression of green fluorescent protein (GFP) in *Tg*(*myf*5(80K):GFP) transgenics matches the dynamic pattern of endogenous *myf5 *expression in cranial muscles**. (A-D) GFP fluorescence is apparent in pectoral fin muscle (pm), dorsal rostral muscle (drm), and hypaxial muscle (hy). (C-E) GFP fluorescence is detected in the occipital somite (os; precursors of sternohyoideus, sh) and some cranial muscles, such as the superior oblique (so) and inferior oblique (io). (F-H) Endogenous *myf5 *transcripts are also detected in cranial muscles, including so and io by whole-mount mRNA *in situ *hybridization. (A,C,F) Side views, rostral to the left, dorsal to the top; (B,D,G) dorsal views, rostral to the left; (E,H) ventral views, rostral to the left. Scale bars: 200 μm.

**Figure 5:**
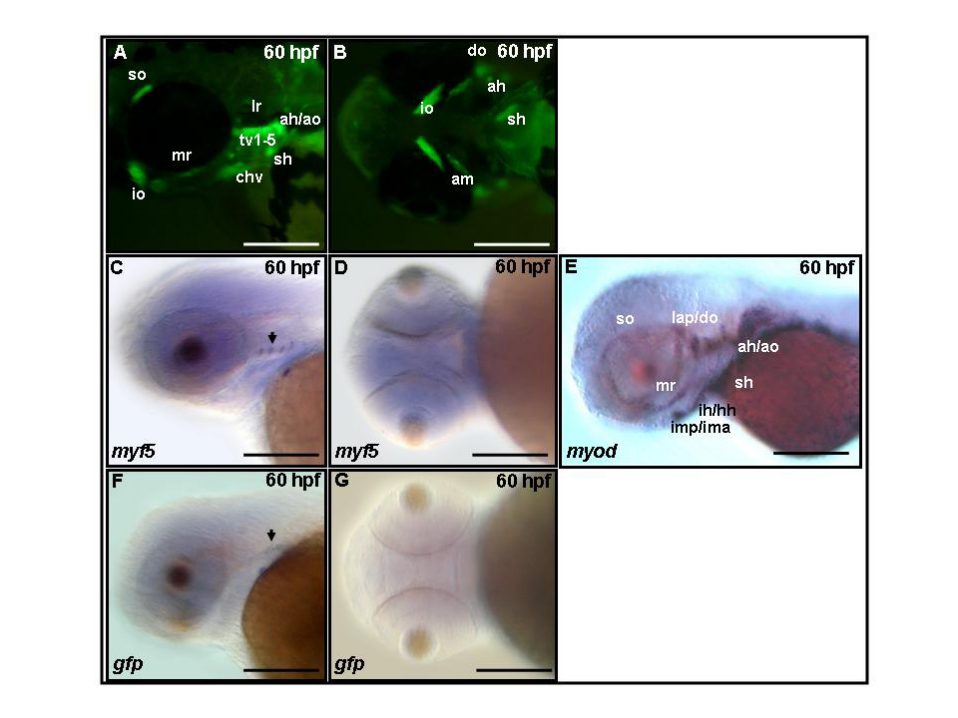
**Green fluorescent protein (GFP) persists in cranial muscles of *Tg*(*myf*5(80k):GFP) transgenics**. (A,B) GFP fluorescence is apparent in adductor hyomandibulae (ah), adductor mandibulae (am), adductor operculi (ao), constrictor hyoideus ventralis (chv), dilatator operculi (do), inferior oblique (io), lateral rectus (lr), medial rectus (mr), sternohyoideus (sh), superior oblique (so), and transverse ventralis (tv1–5) at 60 hours postfertilization (hpf). (C, D) Endogenous *myf5 *transcripts and (F, G) GFP mRNA are restricted to four spots by 60 hpf using whole-mount mRNA *in situ *hybridization. (E) At 60 hpf, *myod *transcripts are detected in most if not all cranial muscles. (A,C,E,F) Side views, rostral to the left, dorsal to the top; (B,D,G) ventral view, rostral to the left. Scale bars: 200 μm in all panels.

By the end of segmentation stages, pectoral fin (pm) and myotomal (m), dorsal rostral myotomal (drm), muscle precursors express GFP (Figs. 4A and 4B). By 36 hpf, in addition to muscle precursors of pm, m, and drm, two eye muscle precursors, the superior oblique (so) and inferior oblique (io), also express GFP (Figs. 4C–4E) and endogenous *myf5 *transcripts (Figs. 4F–4H). At 60 hpf, GFP signals are observed in almost all cranial muscle precursors, including the adductor hyomandibulae (ah), adductor mandibulae (am), adductor operculi (ao), constrictor hyoideus ventralis (chv), dilatator operculi (do), inferior oblique (io), lateral rectus (lr), medial rectus (mr), sternohyoideus (sh), superior oblique (so), and transverse ventralis (tv1–5) (Figs. 5A and 5B). Compared with the broad expression of GFP fluorescence, expression in both GFP and endogenous *myf5 *transcripts is restricted to a tiny spot on each pharyngeal arch at this stage on lateral views (Figs. 5C and 5F, arrows) by using whole-mount *in situ *hybridization. However, either GFP or *myf*5 signals were observed at this stage on ventral views (Figs. 5D and 5G). Thus, we conclude that this inconsistency between GFP mRNA and fluorescence should be due to the stability of GFP that persists even though transcription of *myf5 *has ended. Based on these observations, we propose that most and possibly all cranial muscle precursors transiently express *myf5 *before 60 hpf, although *myod *expression is still evident at this stage (Fig. 5E).

### Proximal element regulates *myf5 *expression in the presomitic mesoderm

**Figure 6:**
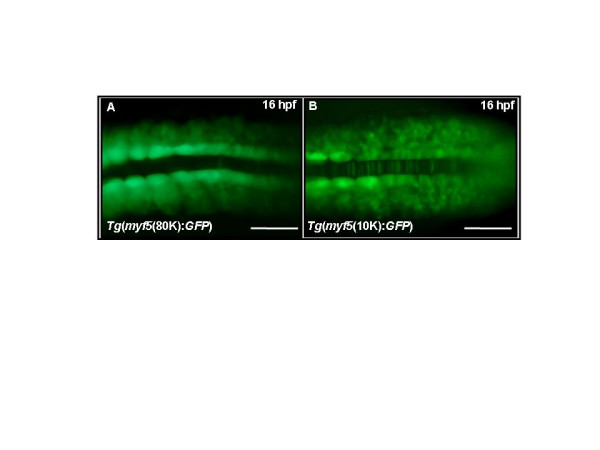
**A proximal element regulates *myf5 *expression in the presomitic mesoderm**. Green fluorescent protein (GFP) fluorescence is detected in the presomitic and somitic mesoderm of transgenic embryos harboring (A) -80 kb, *Tg*(*myf*5(80K):GFP) or (B) -10 kb, *Tg*(*myf*5(10K):GFP)*. Tg*(*myf*5(10K):GFP) embryos also express GFP in the notochord. Dorsal views, rostral to the left. Scale bars: 200 μm.

A unique characteristic of *myf5 *expression, compared with other known MRFs, is its strong and widespread expression in the presomitic mesoderm (Fig. 2B). We used the transgenic lines to identify the regulatory sequences required for this aspect of *myf5 *expression. At 16 hpf, *Tg*(*myf5*(80K)*:GFP*) embryos show strong GFP expression in adaxial cells, presomitic mesoderm, and developing somites (Fig. 6A). Transgenic lines carrying shorter upstream segments, -9977/-1 *Tg*(*myf5*(10K):*GFP*) (Fig. 6B), -6212/-1 *Tg*(*myf5*(6K):*GFP*), -2937/-1 *Tg*(*myf5*(3K):*GFP*), -2456/-1 *Tg*(*myf5*(2.4K):*GFP*) (Table 1), also show strong GFP signals in the presomitic mesoderm. However, in the transgenic line carrying the -290/-1 segment *Tg*(*myf5*(0.3K):*GFP*), the GFP signal in the presomitic mesoderm is weak (Table 1). These results suggest that the minimal enhancer that regulates *myf5 *expression in the presomitic mesoderm is located within the -290/-1 segment.

**Table 1:** Summary of GFP expression domains in different *myf*5:*GFP *transgenic lines

**Transgenic lines**	**Muscles**			**Bones**	**NC**	**SC**	**Eye**	**Somites**	**PSM**
							
	**Head**	**Trunk**	**Fin**						
Tg(*myf5*(80K):*GFP*)	+	+	+	-	-	-	-	+	+
Tg(*myf5*(10K):*GFP*)	-	+	-	-	+	-	-	+	+
Tg(*myf5*(6K):*GFP*)	+	+	-	+	+	+	+	+	+
Tg(*myf5*(3K):*GFP*)	-	-	-	-	+	-	+	+	+
Tg(*myf5*(2.4K):*GFP*)	-	-	-	-	-	-	-	+	+
Tg(*myf5*(0.3K):*GFP*)	-	-	-	-	-	-	-	+	+

NC: notochord; SC: spinal cord; PSM: presomitic mesoderm; +: GFP expression; -: no expression.

### Repressive element located at -80/-10 kb blocks GFP expression in notochord driven by module -2937/-2457

**Figure 7:**
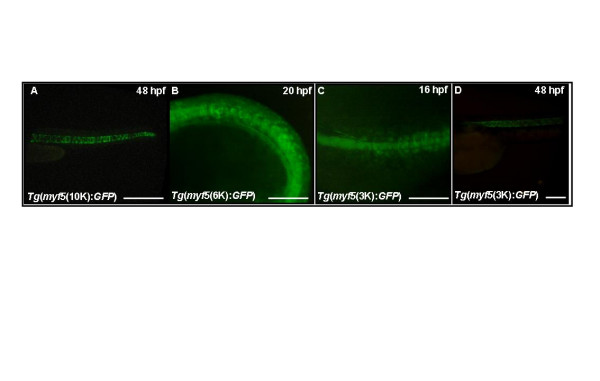
**The *myf5 *upstream region contains modules that repress expression in notochord**. Green fluorescent protein (GFP) fluorescence is detected in the notochord of transgenic embryos harboring (A) -10 kb, *Tg*(*myf*5(10K):GFP), (B) -6 kb, *Tg*(*myf*5(6K):GFP), or (C-D) -3 kb, *Tg*(*myf*5(3K):GFP). Side views, rostral to the left. Scale bars: 400 μm in A; 200 μm in B, C, D.

We observe no GFP signal in the notochords of embryos derived from line *Tg*(*myf5*(80K):*GFP*). However, *Tg*(*myf5*(10K):*GFP*) (Fig. 7A), *Tg*(*myf5*(6K):*GFP*) (Fig. 7B), and *Tg*(*myf5*(3K):*GFP*) (Figs. 7C and 7D) embryos have GFP expression in their notochords, suggesting that an element located within -80/-10 kb normally represses *myf5 *expression in notochord. We also observe no GFP in notochords of embryos derived from lines *Tg*(*myf5*(2.4K):*GFP*) and *Tg*(*myf5*(0.3K):*GFP*) (Table 1), suggesting that the -2937/-2457 segment contains a notochord enhancer element. To test this hypothesis, we constructed and microinjected three different GFP expression plasmids: pEGFPmTATA contains a TATA-box of CMV fused to EGFP, pEGFPm(2937/2457) contains one copy of segment -2937/-2457, and pEGFPm(2457/2937) contains segment -2937/-2457 in the opposite orientation (Additional file 1). Only 5.1% (6 of 104) of pEGFPmTATA-injected embryos are GFP-positive, and in no cases is GFP expressed either in notochord or myocytes (Additional file 1). However, 48.4% of pEGFPm(2937/2457)-injected embryos and 44.5% of pEGFPm(2457/2937)-injected embryos express GFP in both notochord and myocytes (Additional file 1). Thus, the -2937/-2457 segment is a typical, orientation-independent enhancer module for *myf5 *expression in notochord and myocytes.

### Multiple modules regulate *myf5 *expression in the spinal cord, bones, eyes, and olfactory pits

**Figure 8:**
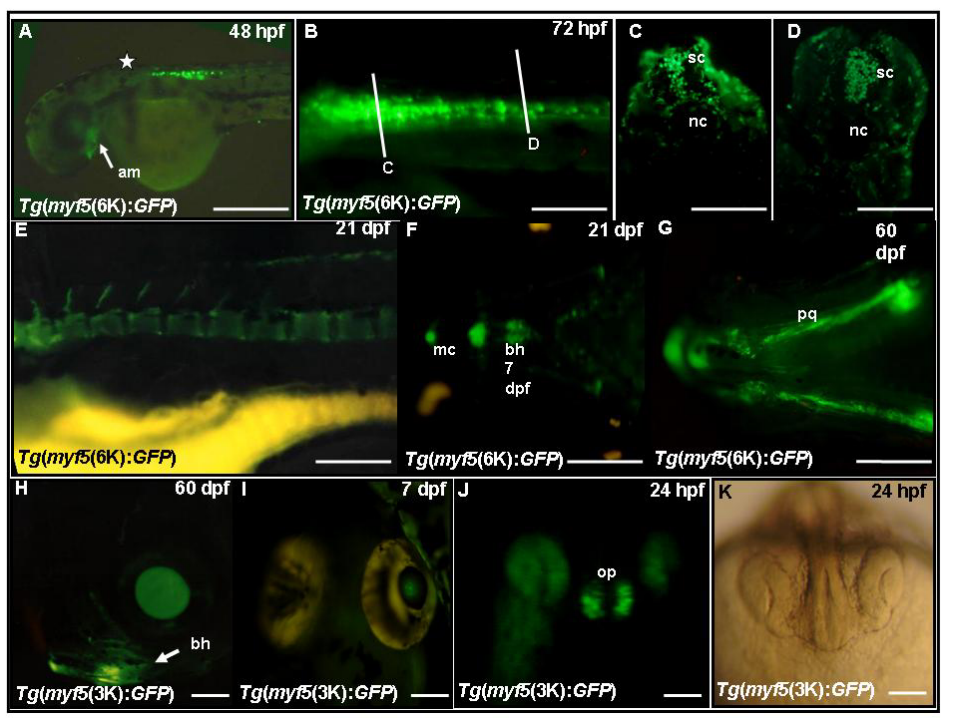
**The *myf5 *upstream region contains modules that regulate expression in spinal cord, bones, eyes and olfactory-pits**. (A,B) Green fluorescent protein (GFP) fluorescence at 48 hours postfertilization (hpf) (A) and 72 hpf (B); the star in A indicates the location of hindbrain. (C,D) Cross-sections along the plane indicated by lines C and D in B, GFP signals are apparent in spinal cord (sc) and surface ectoderm. (E-J) GFP expression is observed in bones at 21 dpf (E and F), in bones at 60 dpf (G and H) in eyes (H and I), and olfactory pits (J). (K) The same embryo as (J) with brightfield illumination. am, adductor mandibulae; bh, basihyal; mc, Meckel's cartilage; n, notochord; op, olfactory pits; pq, palatoquadrate; sc, spinal cord. (A,B,E,H) Lateral views, rostral to the left, dorsal to the top; (F,G) ventral views, rostral to the left; (I-K) frontal views, dorsal to the top. Scare bars: 500 μm in A, E-H; 250 μm in I; 200 μm in B; 100 μm in C, D, J, K.

In embryos derived from *Tg*(*myf5*(6K):*GFP*), GFP fluorescence appears in adductor mandibulae (am) and dorsally caudal to the hindbrain at 48 hpf (Fig. 8A). By 72 hpf, GFP fluorescence is stronger and extends farther caudally (Fig. 8B), including cells of both the surface ectoderm and spinal cord (Figs. 8C and 8D). From 21–60 hpf, the embryos derived from *Tg*(*myf5*(6K):*GFP*) show GFP fluorescence in bone, including the basihyal, sternohyal, and palatoquadrate bones, and Meckel's cartilage (Figs. 8E–8G), and in the eyes (Fig. 8H). We find no significant differences when comparing the GFP expression patterns of the four independent *myf5*:*GFP *lines (6k-9R, -10R, -11R, -16R), indicating that expression of the transgene is unaffected by its chromosomal location. In the head of embryos derived from *Tg*(*myf5*(3K):*GFP*), GFP is expressed primarily in the eyes (Fig. 8I) and olfactory placode (Fig. 8J), suggesting that eye and olfactory enhancers are located within the -2937/-291 segment. Again, we believe that expression of the transgene is unaffected by its chromosomal location, because no significant differences were observed when comparing the GFP expression patterns of the three independent *myf5*:*GFP *lines (3k-18R, -92R, -104R).

## Discussion

### Stable transgenic lines provide greater sensitivity for studies of gene regulation and confirm transient transgenesis studies

**Figure 9:**
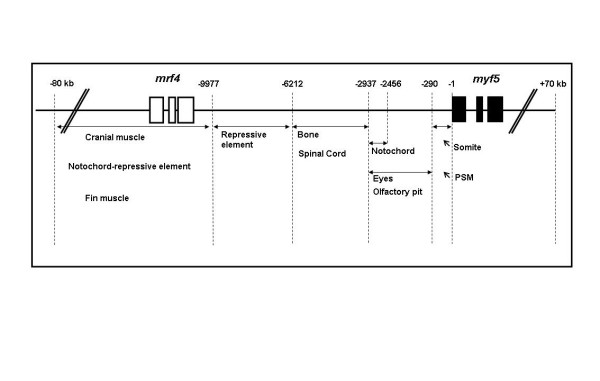
**Multiple upstream modules regulate zebrafish *myf5 *expression**. Thick horizontal lines, blank boxes, and solid boxes represent *myf5 *upstream and downstream regions, *mrf4 *and *myf5*, respectively. Numbers above crossed boxes indicate nucleotide positions relative to the transcription start site of *myf5*.

To understand the complex spatial and temporal regulation of *myf5 *expression, we analyzed the function of the zebrafish *myf5 *promoter using transgenic constructs. Our previous analysis of embryos injected with various lengths of *myf5 *promoter driven reporter genes (transient transgenesis) showed that -9977/-1 [22], -6212/-1 [4], and -2937/-1 [13] produce GFP-positive signals in the notochord, whereas no fluorescence is observed in the notochord when embryos are injected with -2456/-1 [13]. These observations suggest that the notochord-specific element is located in the -2937/-2457 segment, consistent with the germ-line transmission analysis of our present study (Fig. 9).

The regulatory elements for muscle lineage-specific expression, including slow and fast myotomes, are controlled by Hedgehog signaling and myocyte enhancer factor 2 (MEF2) [8,23-25]. We propose that the somite- and myotomal-restricted enhancer element is located within the -290/-1 segment (Fig. 9). Thus, it is important to look for the putative myotomal enhancers, such as Hedgehog-responsive elements and MEF2-binding sites within this segment. After sequence analysis, we find that there are one MEF2-binding (-277/-268) and two Gli-binding (-252/-243 and -196/-183) sites located within the -290/-1 segment. In addition, our previous studies showed that the -82/-62 cassette that contains a binding site for the cognate *trans*-acting factor Foxd3 [15] is able to drive transient expression in somites [14]. On the basis of these observations, we propose that -82/-62 motif is a key element for driving somite specificity, and multiple elements within the -290/-1 segment such as MEF2- and Gli-binding sites are responsible for myotomal expression. Together, these results demonstrate that transient analysis is suitable for rapid identification of putative *cis*-elements, whereas germ-line transmission studies provide great sensitivity and enable confirmation.

### Many *myf5 *regulatory modules are similar between mouse and zebrafish

Few reports have proposed using transgenic animals to study the delicate transcriptional regulation of *myf5*. Only three species (mice, zebrafish, and *Xenopus*) have been documented so far. With limited information on the interactions between *trans*-acting factors and their binding sequences upstream of the *myf5 *gene, the alternative ways to study transcriptional mechanistic conservation are those making comparisons of sequence similarities and genomic organizations of the *myf5 *gene among these three known species. As in zebrafish, *myf5 *expression is regulated by multiple upstream sites in mouse embryos [7,26]. Multiple enhancers are distributed throughout a 90-kb region of the mouse *mrf*4/*myf5 *locus, including a limb enhancer at -58/-48 kb, two head muscle enhancers at -88/-63 and -45/-23 kb, a repressive element at -58/-8.8 kb, a central nervous system-specific enhancer at -0.5/-0.1 kb, a hypaxial myotome enhancer at 0.5/3.5 kb, an epaxial myotome enhancer at -5.6/-4.6 kb, and branchial enhancers at -1.5/-0.5 and 0.5/3.5 kb [6,7,27]. Gene regulation of Myf5 in frog is apparently quite different, because only a relatively short region of flanking DNA (about 1.2 kb) is sufficient to drive endogenous *Xenopus Xmyf5 *expression at gastrula stages. Two negative regulatory elements (an interferon-like regulatory factor and a distal TCF-3 binding site) have been identified in the *Xmyf5 *promoter [10,11]. It seems likely that each species (i.e., mice, zebrafish and *Xenopus*) uses distinct mechanisms, given the developmental differences in timing and signaling.

The genomic organizations of *myf5 *regulatory modules are also conserved between mice and zebrafish. As in mouse, the zebrafish regulatory modules that are responsible for lineage-specific expression of *myf5 *are distributed throughout a large (80-kb) region of the *mrf4*/*myf5 *locus (Fig. 9). The locations and functions of some enhancers are similar to those of mouse *myf5*. For example, the fin muscle enhancer and the cranial muscle enhancer at -80/-10 kb probably correspond to the mouse limb and head muscle enhancers at -88/-23 kb, a repressive one at -10/-6 kb is similar to the mouse repressor at -58/-8.8 kb, and a spinal cord enhancer at -6/-3 kb is similar to the mouse central nervous system-specific enhancer at -0.5/-0.1 kb (Fig. 9).

On the other hand, however, we also identified enhancers that have not been described in mouse, including a bone enhancer at -6/-3 kb, a notochord enhancer at -3/-2.4 kb, and a presomitic mesoderm enhancer at -290/-1 bp (Fig. 9). Epaxial- and hypaxial-specific enhancers have been identified in mouse *myf5 *[6,7,27], but we have not seen muscle lineage-specific enhancers, especially slow and fast muscle enhancers in zebrafish, with the exception of limb and head muscle enhancers. This finding suggests that more transgenic lines that carry shorter elements should be generated, especially deletions within the -80/-10 kb and -10/-6 kb regions. Taken together, we propose that the regulation of zebrafish *myf5 *is more similar to mouse *myf5 *than that of *Xenopus*.

### Excellent experimental materials to study transcriptional regulation on *myf5*

Myf5 is the first member of the MRF family expressed during somitogenesis in zebrafish. Knockdown of *myf5 *in zebrafish results in malformation of somites and brain defects, indicating that trunk and head myogenesis is impaired, underscoring the importance of understanding the regulation of *myf5*. Numerous factors have been implicated as upstream regulators of *myf5*, including extracellular signals, such as *shh*, *wingless *(*wnt*), and fibroblast growth factor (FGF) [28,29]. *Shh*, produced by the notochord and floor plate of the neural tube, and Wnt proteins, produced by the dorsal neural tube and surface ectoderm, have been implicated in the maintenance of mouse *myf5 *[8,21,30,31], *Xenopus Xmyf5 *[32], and zebrafish *myf5 *[33] expression. Together with our previous identification of the cognate *trans*-acting factor, Foxd3 [15], these results demonstrate that the complex spatial and temporal pattern of *myf5 *gene expression is regulated by multiple upstream regulatory modules.

## Conclusion

We generated several transgenic lines of zebrafish that contain various lengths of zebrafish *myf5 *upstream genomic sequence linked to the GFP reporter gene. We demonstrate that the 156-kb genomic sequence (an upstream 80-kb and a downstream 70-kb segment) is able to recapitulate the pattern of endogenous *myf5 *expression. By dissecting this upstream region, we further show that tissue-specific regulatory elements are organized as modules in various regions of the 5'-flanking sequence. These transgenic lines not only provide excellent materials for studying the regulatory mechanism of *myf5*, but they also will facilitate mutant screens to identify novel genes that regulate somitogenesis and more detailed studies to the morphogenesis of somites, presomitic mesoderm, and cranial muscles.

## Methods

### Animals

Embryos were produced using standard procedures [34] and were staged according to standard criteria [35] or by hpf at 28°C. The wild-type line used in this study was AB. Line and gene names follow the zebrafish nomenclature conventions [36].

### BAC library screening

The zebrafish BAC library was obtained from the RZPD [37], and the screening protocols followed the manufacturer's instructions, with minor modifications. The primary BAC library pools were screened by PCR using the zebrafish *myf5 *intron 1-specific primers 1261F (5'-TGTTCATTCACTCATTTTCTTTTCA-3') and 2582R (5'-GCAGTCTTCCTACAATGACAA-3'). The positive clones isolated from the primary pools were further confirmed by screening the secondary pools to isolate a *myf5*-containing BAC clone.

### Pulsed-field gel electrophoresis

DNA from a *myf5*-containing BAC clone was extracted; digested with *Eco*RI, *Hin*dIII, and *Sac*I; and analyzed by 0.8% Agarose pulsed-field gel electrophoresis (PFGE; Biometra). The electrophoresis conditions were 200 volts at 10°C for 24 h with electrode angles at 120°, and rotor speed of 2–6 s. After electrophoresis, the BAC DNA size was analyzed with Kodak 1D image software.

### Bioinformatics

We used the zebrafish *myf5*-specific primers 1261F and 2582R for mapping *myf5 *against the LN54 radiation hybrid (RH) panel. The RH panel was scored according to Hudson *et al*. [38] using the public web site [39]. For screening the *myf5*-containing BAC clone, the junctions of BAC DNA were sequenced using T7 and SP6 primers. These junction sequences and adjacent coding regions were analyzed by BlastN [40], and the location of the *myf5*-containing BAC clone was characterized and named *myf5*(80K).

### Generation of a *myf5*(80K) clone containing the GFP reporter

Plasmid p(*myf5*(80K):GFP) contains the approximately 80-kb region around *myf5 *fused to GFP (Fig. 1A). Basically, we followed the protocols described by Lee *et al*. [17], with some modifications. The cassette used for targeting the *myf5 *locus was amplified from template pZMYP-82E [4] with primers ZMFP-82F (5'-CTCTTAGCTCTGTCCTGGCCA-3') and Kan-817R (5'-ATTTACAAATGAGCAAGCAGTGTGAATAAAGCGTTGGCCTGAGTCGGTCATTTCGAACCCCAG-3') by using Deep Vent (NEB) to carry out PCR at the following conditions: 94°C, 40 s; 58°C, 30 s; 72°C, 150 s; for 35 cycles. PCR products were digested with *Dpn*I to remove template.

For *myf5*(80K) transformation, 1 μg of *myf5*(80K) DNA was used to transform competent cells, *E. coli *DY380. For *myf5*(80K) modification with GFP, putative transformants harboring *myf5*(80K) were isolated and grown in LB medium containing 25 μg/ml of chloramphenicol (CAM) overnight at 32°C with shaking at 150 rpm. Subsequently a 0.1% inoculum was cultured in 50 ml of LB-CAM to an OD_600 _of 0.8. Then, the bacteria were cultured at 42°C for 15 min, transferred to an ice slurry for 30 min, washed 5 times in ice-cold water, and electroporated immediately. Approximately 2 μg of targeting cassette DNA was mixed with the freshly prepared electrocompetent cells of DY380 carrying *myf5*(80K). Electroporation was performed at 1.8 kV, 200 Ohms, 25 μF for 3 × 30 s intervals (Gene Pulser Xcell, BioRad). After electroporation, cells were dispersed on the LB plates with 30 μg/ml of kanamycin and incubated at 32°C overnight. Then, recombinants were picked and checked by PCR using primers ZMFP-117F (5'-TTTGGGTGGGGATCTAGATGGTG-3') and GFP-407R (5'-GTTGCCGTCCT-CCTTGAAGT-3'), and Kan-F (5'-ATGATTGAACAAGATGGATTGC-3') and ZMF-1000R (5'-AGCGAGTTAAGTTTAAAGTCTGACCC-3') to check both integration ends.

### Plasmid constructs for promoter analysis

#### (A) pZMYP-9977E

Following our previously described procedure [4], a 3.7-kb *Sac*I-cut fragment from a *myf5*-positive recombinant bacteriophage was ligated with the *Sac*I-digested plasmid, pZMYP-6212E, in which a -6212/-1 segment of zebrafish *myf5 *was fused with enhanced GFP (EGFP) cDNA. The resulting plasmid, pZMYP-9977E, contained the -9977/-1 segment of zebrafish *myf5 *fused with EGFP cDNA (Fig. 1B).

#### (B) pZMYP-9977E-ITR

A *Sal*I/*Eco*RV fragment from pZMYP-6212E [4] was ligated with a Klenow-filled pGEMT-easy vector (Promega), in which the *Eco*RV site but not the *Sal*I site was retained, to produce an intermediate plasmid, p(6212/1984). Then, a 3.8-kb *Sac*I fragment from a *myf5*-positive phage clone [4] was ligated with *Sac*I-treated p(6212/1984) to produce another intermediate plasmid, p(9977/1984). Finally, a *Sac*I/*Eco*RV fragment cut from p(9977/1984) was ligated with *Sac*I/*Eco*RV-treated pZMYP-6212E-ITR (Fig. 1B). The resulting plasmid, pZMYP-9977E-ITR, contained the -9977/-1 segment of *myf5 *and was flanked with inverted terminal repeats of adeno-associated virus (AAV-ITR) (Fig. 1B).

#### (C) pZMYP-6212E-ITR and pZMYP-2937E-ITR

A *Sal*I/*Age*I fragment from either pZMYP-6212E or -2937E [4] was ligated with a 4.2-kb *Sal*I/*Age*I fragment obtained from pCMV-EGFP-ITR [41]. The resulting plasmids, pZMYP-6212E-ITR and pZMYP-2937E-ITR, contained the -6212/-1 and -2937/-1 segments of zebrafish *myf5*, respectively. Each plasmid was also flanked with AAV-ITR at both ends (Fig. 1B).

#### (D) pZMYP-290E-ITR

Plasmid pZMYP-290E [14] was cut with *Hin*dIII, blunted and then cut with *Age*I. The resulting 0.3-kb fragment was ligated with a 4.2-kb fragment produced by cutting pCMV-EGFP-ITR [41] with *Sal*I, blunting and then cutting with *Age*I. The resulting plasmid, pZMYP-290E-ITR, contained a zebrafish *myf5 *-290/-1 segment and was flanked with AAV-ITR at both ends (Fig. 1B).

#### (E) pEGFPm(2937/2457) and pEGFPm(2457/2937)

For functional identification of the notochord-specific *cis*-element, we used a forward primer (5'-TCTAGAACAGATTCTCATCCAA-3') and a reverse primer (5'-AACTGCACACTGGAGATTCATAAG-3') to generate module -2937/-2457. This module was ligated with pGEM T-Easy vector and then treated with *Eco*RI to produce the insert. *Eco*RI-cut pEGFPmTATA, which contained a minimal TATA-box of cytomegalovirus (CMV) promoter for the EGFP gene [14], was ligated with one copy of the *Eco*RI-cut module -2937/-2457 to generate pEGFPm(2937/2457). Plasmid pEGFPm(2457/2937), containing one copy of module -2457/-2937, was also constructed.

### DNA preparation for microinjection and transient GFP expression

The procedures of microinjection and transient GFP detection were described by Chen *et al*. [4], except that we observed the GFP expression of transgenic embryos hourly, especially from 6 to 36 h.

### Identification of germ-line transmission

All GFP-positive embryos at 24 h were raised to adulthood. Transgenic founders (F0) were mated with wild-types individually to confirm that they could transmit the BAC through the germ line. At least 200 embryos from each pair were examined for GFP fluorescence. After screening, GFP-positive F1 embryos were raised to adulthood and crossed with wild-type adults to generate the heterozygous F2 generation. GFP-positive F2 individuals were then crossed to each other to generate homozygous F3 fish that produced 100% GFP-positive F4 offspring.

### Antibody labeling

Antibody labeling was performed as previously described, with minor modifications [42]. Embryos were fixed in 4% paraformaldehyde in phosphate buffered saline (PBS, pH 7.0) for 4 h at room temperature, or overnight at 4°C. Then, embryos were washed in 0.1 M PBS twice for 15 min each, soaked in 100% acetone at -20°C for at least 10 min, and rehydrated with 0.1% (v/v) Tween 20 in PBS 3 times for 15 min each. After rehydration, the embryos were treated with PBS containing 5% goat serum albumin and subjected to immunofluorescence labeling. To detect zebrafish slow muscle fibers, the F59 monoclonal antibody (1:10; Hybridoma Bank) was used with Alexa Fluor 568 rabbit anti-mouse IgG (1:200; Molecular Probes) as the secondary antibody.

### Whole-mount *in situ *hybridization, cryosection and fluorescence observation

The procedures of cryosectioning and whole-mount *in situ *hybridization were described by Chen and Tsai [43], except that embryos from 7.5 to 60 h were used. Transgenic embryos were observed hourly, especially from 1 to 14 hpf, under a stereo fluorescence dissecting microscope (MZ12, Leica) equipped with GFP and DsRed filter cubes (Kramer Scientific). Photographs were taken with an S2 Pro digital camera (Fuji).

## Authors' contributions

Y.H.C. designed the study, carried out the experiments, analyzed the data, and drafted the manuscript. Y.H.W. cloned the *myf5*-containing BAC clone, generated the *Tg*(*myf5*(80K):*GFP*) line, and participated in the coordination of the study and in drafting the manuscript. M.Y.C. carried out the whole-mount *in situ *hybridization and antibody labeling experiments. C.Y.L. generated the *Tg*(*myf5*(3K):*GFP*) and *Tg*(*myf5*(0.3K):*GFP*) lines. C.W.W. identified the notochord-restricted element. M.W and H.J.T. gave discussion and helped in editing the manuscript. H.J.T. was a principal investigator of this project.

## Supplementary Material

Additional file 1 **Cassette -2937/-2457 directs the GFP expression in notochord**. Upper left: Schematic illustration of microinjected plasmids pEGFPmTATA, pEGFPm(2937/2457), and pEGFPm(2457/2937). Right: The calculation of total expression rates, notochord- and myocyte-specific expression rates, and nonspecific expression rates are described before [14]. Bottom: Embryos were photographed under fluorescent light. In pEGFPm(2937/2457)-injected zebrafish, EGFP signals appeared as bars with sharp edges (myocyte-specific) and squares (notochord-specific). Scale bar: 200 μm. Click here for file
