# Supplementary figures and images for: Multiple upstream modules regulate zebrafish myf5 expression
Source: BMC Dev Biol. 2007 Jan 3;7:1. doi: 10.1186/1471-213X-7-1 (PMC1769357; doi:10.1186/1471-213X-7-1)

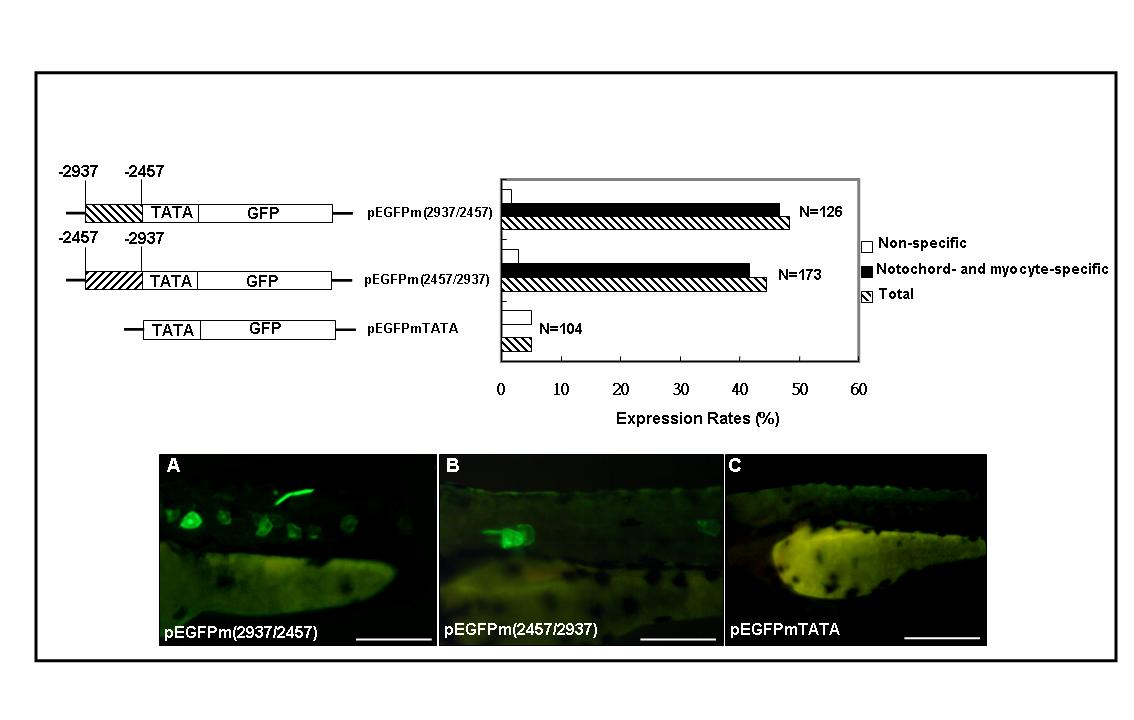

Supplement: Additional file 1 — Cassette -2937/-2457 directs the GFP expression in notochord. Upper left: Schematic illustration of microinjected plasmids pEGFPmTATA, pEGFPm(2937/2457), and pEGFPm(2457/2937). Right: The calculation of total expression rates, notochord- and myocyte-specific expression rates, and nonspecific expression rates are described before [14]. Bottom: Embryos were photographed under fluorescent light. In pEGFPm(2937/2457)-injected zebrafish, EGFP signals appeared as bars with sharp edges (myocyte-specific) and squares (notochord-specific). Scale bar: 200 μm. [file 1471-213X-7-1-S1.jpeg]
